# The Methylation and Expression of LINC00511, an Important Angiogenesis-Related lncRNA in Stomach Adenocarcinoma

**DOI:** 10.3390/ijms26052132

**Published:** 2025-02-27

**Authors:** Zhiying Li, Yingli Chen, Yuanyuan Zhao, Qianzhong Li

**Affiliations:** 1 Laboratory of Theoretical Biophysics, School of Physical Science and Technology, Inner Mongolia University, Hohhot 010021, China; lzy15174801530@163.com (Z.L.);; 2 The State Key Laboratory of Reproductive Regulation and Breeding of Grassland Livestock, Inner Mongolia University, Hohhot 010021, China

**Keywords:** angiogenesis, long non-coding RNA, DNA methylation, stomach adenocarcinoma

## Abstract

Stomach adenocarcinoma (STAD) has high incidence and mortality rates. Long non-coding RNAs (lncRNAs) and angiogenesis are closely related to the pathogenesis and metastasis of STAD. Recently, emerging evidence demonstrated that DNA methylation plays crucial roles in the development of STAD. This study explored the relationship between DNA methylation and the abnormal expression of angiogenesis-related lncRNAs (ARlncRNAs) in stomach adenocarcinoma, aiming to identify prognostic biomarkers. Moreover, a Cox analysis and Lasso regression were used to establish an ARlncRNA feature set related to angiogenesis. The prognostic model was evaluated by using a Kaplan–Meier (KM) analysis, ROC curves, and nomograms. Based on the identified 18 key ARlncRNAs, a prognostic predictive model was constructed. In addition, a specific ARlncRNA with abnormal methylation in the model, LINC00511, showed significant differences in expression and methylation across different subgroups. The methylation and expression of LINC00511 were analyzed by a correlation and co-expression analysis. The correlation analysis indicated that promoter methylation may improve LINC00511 expression. Further analysis found 355 mRNAs co-expressed with LINC00511 which may interact with 6 miRNAs to regulate target gene expression. The abnormal methylation of LINC00511 could significantly contribute to the progression of stomach adenocarcinoma.

## 1. Introduction

Stomach cancer (SC) ranks the fifth position in incidence and the fourth in cancer-related deaths among the most prevalent malignant tumors globally [1]. Stomach adenocarcinoma (STAD), as its most common subtype, accounts for 95% of all stomach cancer cases [2]. However, the early clinical manifestations of stomach cancer are often more insidious, leading to a delay in diagnosis, with about 60% of patients already having local or distant metastases at the time of diagnosis [3]. Tumor angiogenesis is a crucial element in the progression of tumors and significantly contributes to their growth and metastasis [4]. During tumor growth, because of the swift increase in cancer cell numbers, the oxygen and nutrition supply in the tumor is insufficient, so new blood vessels are needed to supply sufficient nutrients and oxygen [5]. The stomach, as the reservoir of the digestive tract, has a rich and complex blood supply. In stomach cancer, the blood vessels have abnormal and irregular morphology, an increase in angiogenesis, and a high density of new blood vessels [6]. Studies have shown that blood vessels in SC often exhibit invasive growth, making it more likely to spread in the body and form distant metastases [7].

Research has shown that long non-coding RNAs (lncRNAs) have a vital impact on the development of cancer and play many functions related to cancer characteristics, particularly inducing angiogenesis to promote tumor metastasis [8]. Therefore, more and more scholars pay attention to the exploration of angiogenesis-related lncRNAs (ARlncRNAs) [9,10,11]. Based on 119 angiogenesis-related genes (ARgenes) in the MSigDB database, Han et al. constructed a prognostic risk model for STAD ARlncRNAs [9]. Xu et al. performed the clustering analysis of STAD patients using 36 ARgenes in the MSigDB database, and identified 6 ARlncRNAs and experimentally validated gene expression [10]. Based on AR genes from the GeneCard database, four ARlncRNAs were identified as potential prognostic biomarkers for lung adenocarcinoma patients by using the Cox regression analysis. The reliability of the model was further validated by using Kaplan–Meier (KM) survival curves and receiver operating characteristic (ROC) curves [11]. However, tumor angiogenesis is an extremely complex mechanism involving multiple biological processes [12]. Certain epigenetic changes have been noted to affect the expression of ARgenes within tumor cells [13].

DNA methylation plays a critical role in regulating gene expression. Growing evidence has shown that the relationship between DNA methylation and lncRNAs is significant in the occurrence and progression of diseases. lncRNAs as regulators affect the methylation of associated genes, thereby leading to the occurrence and development of tumors [14]. For example, the lncRNA ANRIL regulates the methylation of PLZF and inhibits the growth of stomach cancer cells [15]. In addition, DNA methylation can also regulate the expression of lncRNAs. Studies have shown that the hypermethylation of promoter and enhancer regions in lncRNA MEG3 are linked to the low survival rate of patients with gastric cardia adenocarcinoma (GCA) [16].

**Figure 1:**
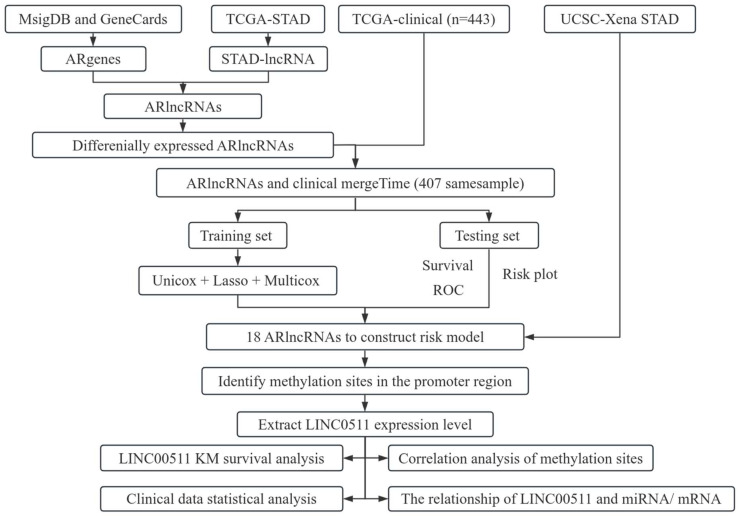
Flowchart of the work.

In this article, we investigated the relationship between angiogenesis-related lncRNAs (ARlncRNAs) and DNA methylation in stomach adenocarcinoma (STAD). We integrated ARgenes in the MSigDB and GeneCard database, and screened out ARlncRNAs. Combined with the clinical data of STAD patients provided in TCGA database, 18 critical ARlncRNAs were identified for the prognosis of STAD patients. In addition, we explored the methylation sites in promoter regions of 18 ARlncRNAs and found 11 methylation sites in the promoter region of LINC00511. We further examined the expression and methylation of LINC00511 in STAD. The workflow of our study is shown in Figure 1.

## 2. Results

### 2.1. Analysis of ARlncRNAs in STAD

#### 2.1.1. Construction of ARlncRNAs Prognostic Model

**Figure 2:**
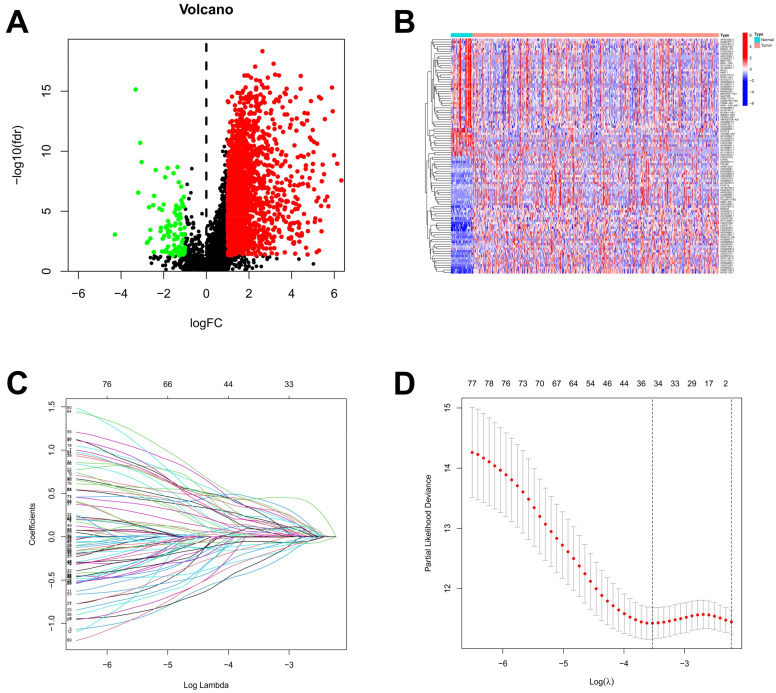
Identification of ARlncRNAs in patients with STAD. (**A**) The volcano plot of 2108 differentially expressed ARlncRNAs; (**B**) the expression profiles of differentially expressed ARlncRNAs in tumor and normal; (**C**) the trajectory of the coefficient of the independent variable; and (**D**) Lasso coefficient spectrum.

Based on the lnRNAs annotated in the Genecode website, 16,876 lncRNAs were extracted from the TCGA dataset. Through a co-expression analysis, 4664 out of 16,876 lncRNAs were identified and designated as ARlncRNAs in STAD. The 2108 differentially expressed ARlncRNAs between STAD patients and normal samples from 4664 lncRNAs were identified, including 2007 up-regulated ARlncRNAs and 101 down-regulated ARlncRNAs (Figure 2A). The visualization of the expression patterns of the 50 differentially expressed ARlncRNAs that were most significantly up-regulated and down-regulated was shown in Figure 2B. There was a significant difference in the expression of ARlncRNAs between STAD tumor tissues and normal stomach tissues. A lasso regression analysis was performed on the 80 differentially expressed ARlncRNAs associated with prognostic survival ARlncRNAs from a univariate Cox analysis for in-depth contraction and selection (Figure 2C,D).

#### 2.1.2. Assessment and Verification of Prognostic Models

**Figure 3:**
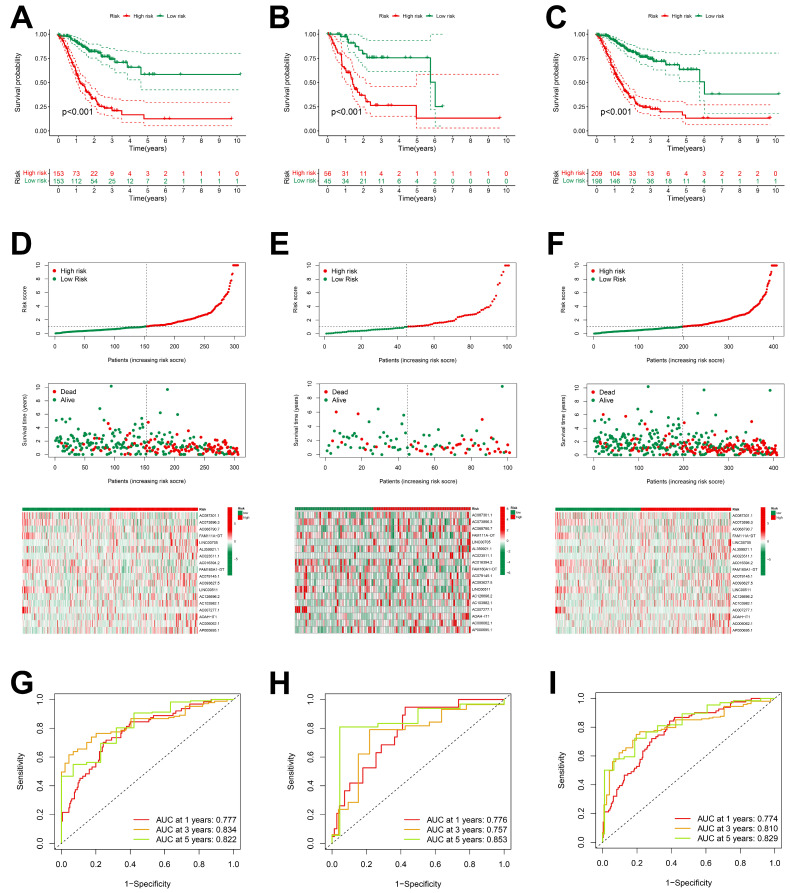
Prognostic characteristics of 18 ARlncRNAs in the TCGA. (**A**–**C**) Survival curves for STAD patients categorized into high-risk and low-risk groups across the training group, validation group, and overall dataset; (**D**–**F**) risk scores, survival status, and risk heatmaps of three groups of STAD patients; and (**G**–**I**) ROC curves of three groups of STAD patients.

The analysis of the Kaplan–Meier survival curves in the training group indicated that patients of the low-risk group exhibited a more favorable prognosis compared to those of the high-risk group (Figure 3A–C). Risk curves and survival status profiles shown that the risk of death increased with risk score. We found that the expression levels of these 18 differentially expressed ARlncRNAs changed with the risk score (Figure 3D–F).

The ROC curve indicated that all the groups exhibited a higher area under the curve (AUC > 0.75) at 1, 3, and 5 years, indicating that the risk scoring model had a high accuracy (Figure 3G–I). The higher accuracy of our prognostic model was also verified for the validation set and TCGA dataset.

#### 2.1.3. $R_{s}$ an Independent Prognostic Factor for STAD

**Figure 4:**
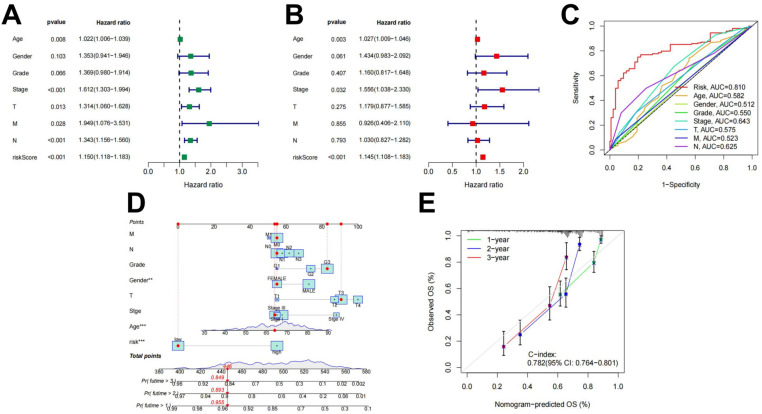
Independent prognostic value of $R_{s}$. (**A**) Results of the univariate Cox independent prognostic analysis; (**B**) results of the multivariate independent prognostic analysis; (**C**) timeROC curve; (**D**) a nomogram designed to predict overall survival (OS) in patients with STAD; and (**E**) C-index curves for predicting 1-, 2-, and 3-year survival rates (** p<0.01, *** p<0.001).

Univariate and multivariate independent prognostic analyses were used to evaluate the independent predictive abilities of the 18 differentially expressed ARlncRNAs signature for STAD patients. Despite the univariate cox analysis showing that risk score, the Stage and *N* stage (*p* < 0.001) were associated to the prognosis of STAD patients (Figure 4A), and the multivariable Cox analysis indicated that only the risk score (*p* < 0.001) served as an independent prognostic factor (Figure 4B). Clinical characters and prognostic models combined with the ROC curve analysis obtained the 0.810 AUC value for the risk score (Figure 4C). Using the constructed $R_{s}$ to predict patient survival results is superior to other clinical characters. Nomograms constructed by using independent prognostic factors as variables (Figure 4D) and calibration curves (Figure 4E) indicated that the predicted results were consistent with actual survival in STAD patients.

#### 2.1.4. Analyses of Methylations in ARlncRNAs in Prognostic Models

The analyses of promoter regions for 18 differentially expressed ARlncRNAs revealed that only 2 ARlncRNAs promoter regions had methylation sites, 2 methylation sites were located in the LINC00705 promoter region, and 11 methylation sites were located in the LINC00511 promoter region. The differential expression and methylation survival analyses of the two ARlncRNAs showed that the expression of LINC00705 and the methylation level had little effect on the survival of STAD patients, but LINC00511 was significantly associated with overall survival (OS) in STAD patients.

Based on the above analysis results, the unique characteristics of LINC00511 were identified. Furthermore, the abnormal expression of LINC00511 has been observed in several other types of cancer besides STAD. Zhu et al. found that LINC00511 was highly expressed in non-small-cell lung carcinoma [17]. Lu et al. observed that LINC00511 expression was higher in cervical cancer than in adjacent normal tissues [18]. Wang et al. reported that LINC00511 competitively interacts with miR-424, thereby promoting proliferation and metastasis in hepatocellular carcinoma [19]. LINC00511 expression was found to be higher in multiple cancers and high expression was associated with tumor progression. Our results indicated that high LINC00511 expression in cancer may come from the abnormal methylations of its promoter regions.

### 2.2. LINC00511 Expression and Methylation Analysis

#### 2.2.1. LINC00511 Expression Correlates with Survival in STAD Patients

**Figure 5:**
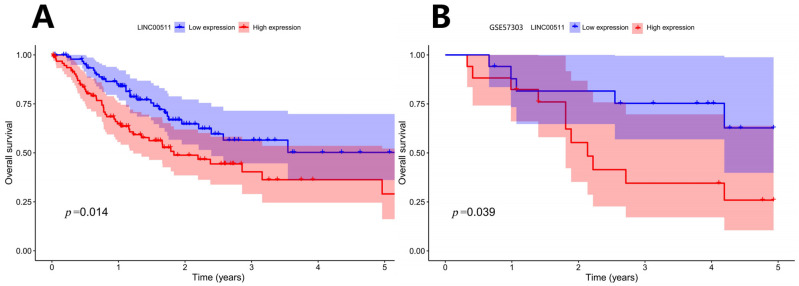
Kaplan–Meier survival curve: OS curve by high and low expression of LINC00511. (**A**) TCGA-STAD Kaplan–Meier survival curve; (**B**) GSE57303 Kaplan–Meier survival curve.

Patients were ranked according to the expression level of LINC00511 from high to low. The patients with the top 25% were defined as high-expression samples and the patients with the bottom 25% were defined as low-expression samples. Kaplan–Meier survival analyses were shown in Figure 5A. The results indicated that patients with a lower expression of LINC00511 had better OS. To validate this finding, we applied the same method to classify LINC00511 expression into high and low groups in the independent sample GSE57303 and performed a survival analysis. The results showed that patients in the low expression group of LINC00511 had significantly better OS compared to those in the high expression group (Figure 5B), further confirming the association between a low expression of LINC00511 and improved prognosis in cancer patients. By comparing LINC00511 expression between STAD and adjacent normal tissues from TCGA, we found that the expression of LINC00511 was higher in tumors than normal tissues (*p* = 1.5 × 10^−8^). This result is consistent with the high expression of LINC00511 in a variety of cancers [17,18,19]. Therefore, inhibiting LINC00511 expression may be helpful for the treatment of STAD.

#### 2.2.2. Analysis of Abnormal DNA Methylation

**Figure 6:**
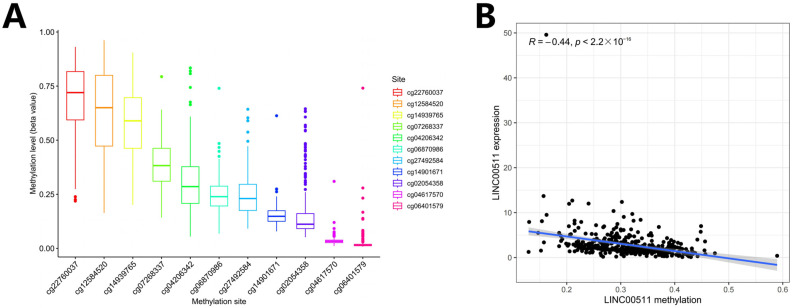
DNA methylation and Spearman correlation analysis. (**A**) Ranking of methylation levels at 11 CpG sites from high to low; (**B**) the relationship between the expression levels of LINC00511 and the methylation levels of CpG sites.

We computed the methylation values of 11 DNA methylation sites in the LINC00511 promoter region (Figure 6A) and the Spearman correlations between the LINC00511 expression levels and the methylation levels of 11 methylation sites. The result indicated that methylation levels of 10 sites were negatively correlated with the expression level of LINC00511, i.e., hypomethylation with high expression (Figure 6B, Figure S1). The methylation level of cg27492584 was most significantly negatively correlated with the expression level of LINC00511 (Figure S1H) (R = −0.53, *p* < 2.2 × 10^−16^). These results indicate that the occurrence of STAD may be due to the hypomethylations at 10 sites in the LINC00511 promoter region which result in the overexpression of LINC00511.

#### 2.2.3. Clinical Data Statistics of the Expression of LINC00511 for STAD Patients

**Figure 7:**
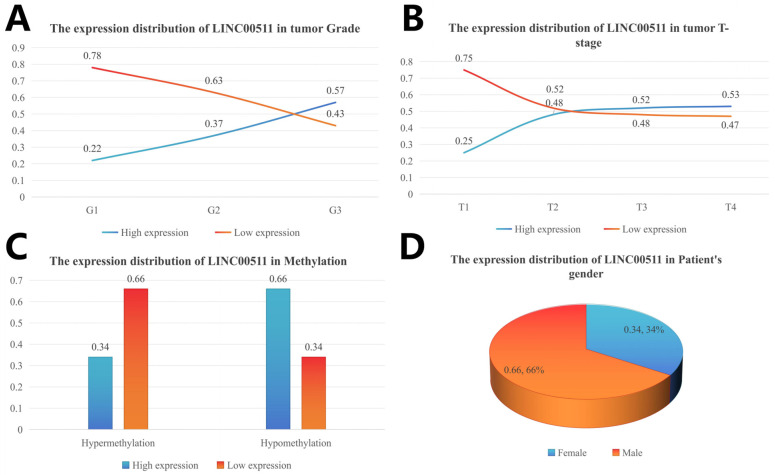
Distribution of LINC00511 expression in clinical characteristics of stomach adenocarcinoma patients. (**A**) Grade; (**B**) tumor T-stage; (**C**) methylation; and (**D**) patient’ s gender.

The clinical characteristics were divided into subgroups and the number of people in each subgroup was calculated. The number of patients with high and low expression in each subgroup was calculated according to the median value of gene expression, and the percentage of high/low expression in the clinical subgroup was calculated. The distribution of different clinical characteristics is shown in Figure 6. The high expression levels of LINC00511 were associated with high-grade tumors. A higher percentage (57%) of G3 tumors had high LINC00511 expression, compared to G1 and G2 tumors (Figure 7A). Similar trends were also observed for the T staging of tumors, with the percentage of patients with a high expression of LINC00511 increasing with increasing tumor volume (Figure 7B). More patients with high methylation had low LINC00511 expression compared to those with a low-methylation tumor (Figure 7C). In addition, the number of male patients is much higher than that of females (Figure 7D), which might be associated to the eating habits and behaviors of males [20].

**Figure 8:**
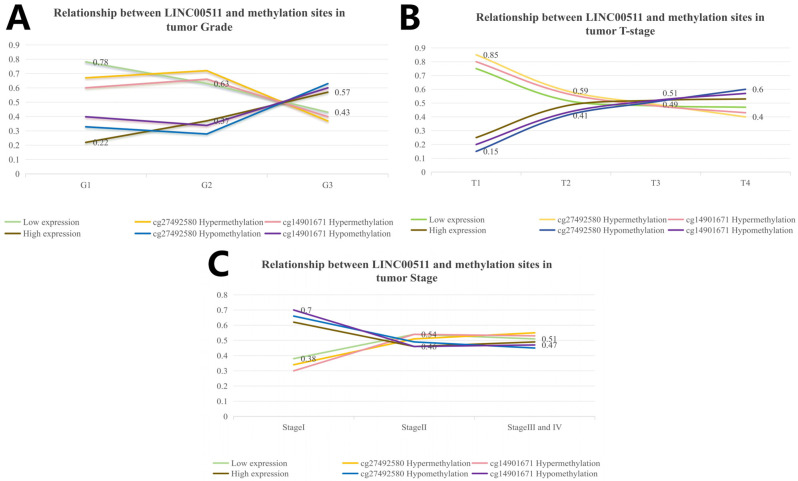
Relationship between LINC00511 and methylation sites in clinical characteristics of stomach adenocarcinoma patients. (**A**) Grade; (**B**) tumor T-stage; and (**C**) tumor Stage.

Using the above calculation method, we analyzed the correlation between DNA methylation in 10 methylation sites and clinical characteristics in stomach adenocarcinoma. Our study showed that hypomethylation of cg27492584 was significantly associated with high-grade tumors (*p* = 1.4 × 10^−9^). In G3 tumors, the percentage of cg27492584 hypomethylation was higher (63%) and the trend of cg27492584 hypermethylation was consistent with the trend of low LINC00511 expression. By calculating the distribution of methylation levels of cg14901671 (*p* = 1.4 × 10^−9^) in clinical characteristics, we obtained the same conclusion (Figure 8A). The reliability of this result was also validated by tumor T-staging (Figure 8B) and disease Stage (Figure 8C), more patients in T4 had cg14901671 hypermethylation (*p* = 0.012) and cg27492584 hypermethylation (*p* = 0.001) compare to those in T1, T2, and T3.

Clinical correlation analyses showed that methylation levels of LINC00511 varied somewhat more significantly across clinical subgroups, compared to the expression levels of LINC00511 across different clinical subgroups. The results showed that the methylation levels of LINC00511 were different among the age, grade, stage, and T-stage subgroups (Figure S2).

#### 2.2.4. The Relationship of LINC00511 and miRNA/mRNA

**Figure 9:**
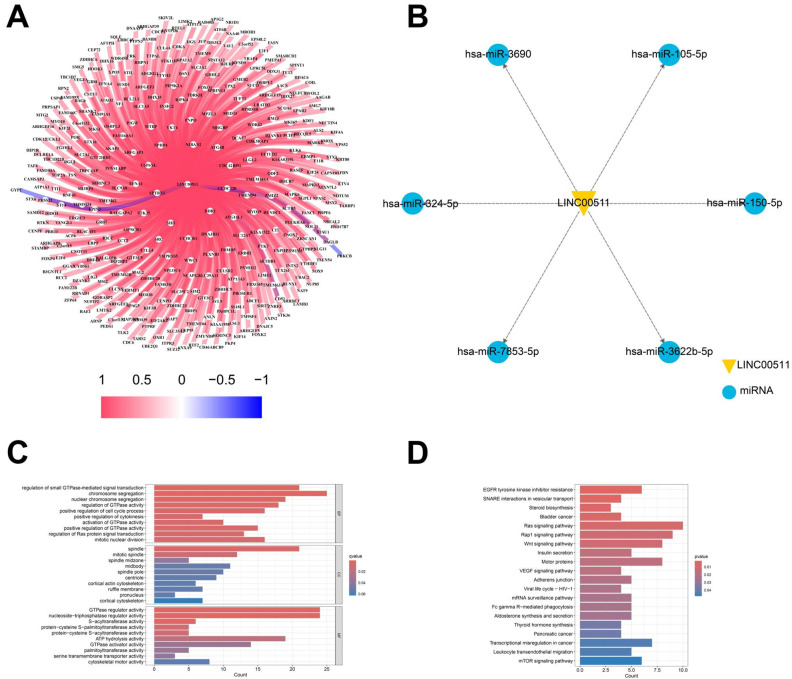
Target gene analysis of LINC00511. (**A**) The relationship of LINC00511 and mRNA; (**B**) the relationship of LINC00511 and miRNA; (**C**) GO enrichment analysis of 355 mRNAs; and (**D**) KEGG pathway of 355 mRNAs.

LncRNAs often exert their functions through complementary binding with other RNA transcripts [21]. We analyzed the correlation of LINC00511 expression with mRNAs in TCGA. We identified 355 mRNAs targeted by LINC00511 through a co-expression analysis. The correlation analysis of these mRNAs and LINC00511 expression showed that 352 mRNAs were positively regulated with LINC00511 (Figure 9A). To further understand the important biological functions associated with LINC00511, we performed GO and KEGG enrichment analyses. The GO enrichment analyses revealed that these genes were predominantly enriched in the regulation of GTPase-mediated signaling, chromosome segregation, and the regulation of some enzyme activities (Figure 9C). The KEGG pathway showed that these genes were mainly involved in the traps of tyrosine kinase inhibitor resistance, vesicle transport interactions, Ras signaling pathway, and bladder cancer-related pathways (Figure 9D). Bladder metastasis from stomach cancer has also been reported as a distinct clinical entity in recent years [22]. LINC00511 is expected to be a prognostic marker for bladder metastasis in stomach cancer.

In addition to binding to mRNAs, lncRNAs can also act as miRNA “sponges”, binding and regulating miRNA activity, thereby affecting miRNA-mediated target gene expression. The starBase database was utilized to identify the potential target miRNAs of LINC00511, resulting in the identification of 6 miRNAs: hsa-miR-3690, hsa-miR-105-5p, hsa-miR-7853-5p, hsa-miR-150-5p, hsa-miR-3622b-5p, and hsa-miR-324-5p. It is worth noting that both hsa-miR-105-5p and hsa-miR324-5p have been previously associated with the development of various cancers (Figure 9B). In these results, it has been experimentally verified that LINC00511 can promote the proliferation, migration, and invasion of esophageal cancer cells by regulating miRNA-150-5p [23]. This finding further supports our prediction that LINC00511 may be involved in cancer-related miRNA pathways.

## 3. Discussion

In this study, we integrated two data groups in stomach adenocarcinoma (STAD) into new ARgenes datasets. A prognostic model comprising eighteen ARlncRNAs was constructed by the Cox regression analysis of ARlncRNAs associated with STAD, and its prognostic performance was validated by patient survival time, survival status, and clinical characteristics. The results of the Kaplan–Meier survival curve and risk curves analyses showed that as the risk score increased, so did the patient’s risk of death. This finding not only helps to differentiate the high-risk from the low-risk patients, but also allows clinicians to identify high-risk individuals for early intervention and monitoring. The ROC curves analyses showed that the area under the curves (AUC) of the model were relatively high at 1, 3, and 5 years (AUC > 0.75). In addition, the validation results of the independent group and TCGA dataset further confirmed the high accuracy of the prognostic model, which showed that the model may be an effective prognostic assessment tool in clinical practice. Combining the ARlncRNA risk score with clinical characteristics, the AUC value of the ROC curve was 0.810, indicating that the model is superior to traditional clinical factors in predicting survival and can guide clinical treatment decisions.

Subsequently, a key ARlncRNA, LINC00511, was identified by the DNA methylation analysis in STAD. Research has found that LINC00511 is highly expressed in stomach adenocarcinoma and is associated with poor prognosis in patients. LINC00511 can affect gene expression through multiple mechanisms. The co-expression analysis with TCGA data revealed that LINC00511 is significantly correlated with 355 mRNAs, most of which were positively regulated. In addition, LINC00511 can act as a miRNA “sponge” to bind to and regulate miRNA activity, thus affecting the expression of target genes. By using the starBase database, we found that LINC00511 interacts with several cancer-related miRNAs (e.g., hsa-miR-105-5p and hsa-miR-324-5p), which may indirectly regulate gene expression by modulating miRNA action. It has been experimentally found that the overexpression of LINC00511 promoted the proliferation, migration, and invasion of gastric cancer cells [24].

In fact, the role of LINC00511, an important oncogenic lncRNA, in cancer has been confirmed in multiple studies, especially in its interactions with miRNAs. Qian et al. identified the synergistic effect of LINC00511 and miRNA-625-5p in colon cancer by transfecting colon cancer cells with oligonucleotides [25]. Their experiments showed that knocking down LINC00511 or restoring miR-625-5p both led to a decrease in the proliferation, migration, and invasion abilities of colon cancer cells. Further studies revealed that miR-625-5p negatively regulated the expression of WEE1 by binding to the 3′UTR region of WEE1. It was ultimately determined that the down-regulation of LINC00511 impeded colon cancer progression by restoring miR-625-5p and silencing WEE1 [25].

Recent research has indicated that DNA hypomethylation in promoter regions leads to the up-regulation of LINC00511 expression, thereby promoting the growth and metastasis of breast cancer tumors [26]. Our findings indicated that the level of methylation of LINC00511 was inversely related to its expression (R = –0.44, *p* < 2.2 × 10^−16^), suggesting that DNA hypomethylation may also contribute to the up-regulation of LINC00511 expression in STAD. Specifically, we focused on two methylation sites, cg27492584 and cg14901671, and observed that their hypomethylation in stomach cancer samples is closely associated with the high expression of LINC00511. This finding indicates that these abnormal methylation sites may play a significant role in the epigenetic regulation of LINC00511 in stomach cancer. Moreover, this result further supports the potential involvement of epigenetic modifications in the regulation of LINC00511 expression, which was consistent with previous studies.

However, due to limitations in conditions, our research remains at the level of theoretical analysis. The prognostic model was only validated on an internal validation set, and can be evaluated by experimental validation in the future. Especially if the model can identify different subtypes of stomach cancer and predict their clinical outcomes, it will have great clinical application value.

Although the model primarily focuses on STAD, its applicability in other SC subtypes can be explored by adjustments and optimizations. For example, finding abnormal DNA methylation sites associated with stomach cancer: In some stomach cancer patients, cancer development is closely associated with the changes in DNA methylation levels in key methylation sites. Future research can explore whether the model can recognize the clinical and molecular characteristics of this particular subtype, thereby achieving early diagnosis and personalized treatment.

## 4. Materials and Methods

### 4.1. Screening ARlncRNAs

Transcriptome sequencing data of long non-coding RNA (lncRNA), messenger RNA (mRNA), and corresponding clinical data of patients diagnosed with stomach adenocarcinoma (STAD) were obtained from The Cancer Genome Atlas (TCGA) (https://portal.gdc.cancer.gov/, accessed on 23 October 2023) database. After removing samples with the survival duration of fewer than 30 days and those with an unknown survival status, a total of 407 TCGA-STAD patients were included in this study. Based on the lnRNAs annotated in the Genecode website (https://www.gencodegenes.org/, accessed on 23 October 2023), 16,876 lncRNAs were extracted from the TCGA dataset. Angiogenesis-related genes (ARgenes) were collected from the GeneCard (https://www.genecards.org/, accessed on 23 October 2023) database and the Molecular Signatures Database (MSigDB) (http://software.broadinstitute.org/gsea/msigdb/, accessed on 23 October 2023) database. A total of 151 ARgenes with a score greater than 5 and belonging to protein coding were downloaded from the GeneCard database, and 415 ARgenes with angiogenic function were downloaded from the MSigDB database. Subsequently, the genes downloaded from the two databases were combined to obtain 482 ARgenes. Among them, 476 ARgenes were characterized in STAD.

Through a co-expression analysis (|R| > 0.4, *p* < 0.001), 4664 out of 16,876 lncRNAs were identified and designated as ARlncRNAs in STAD. The “limma” package in the R software was used to identify 2108 ARlncRNAs that were significantly differentially expressed between STAD patients and normal samples from 4664 lncRNAs, with selection criteria set at FDR < 0.05 and |log_2_FC| ≥ 1.

### 4.2. Identification of Prognostic Features

The “caret” package was used to randomly divide 407 STAD samples into a training group and verification group in a 3:1 ratio. In the training group, the differentially expressed ARlncRNAs were screened by a univariate Cox analysis, and 80 prognosis-related differentially expressed ARlncRNAs were obtained. A Lasso regression analysis and multivariate Cox analysis were conducted on the differentially expressed ARlncRNAs, and 18 differentially expressed ARlncRNAs were obtained as a new prognostic biomarker. The prognostic model was constructed by using the expression values of ARlncRNAs. The Risk score ($R_{s}$) of the prognostic model was defined as follows:

(1) $$R_{s}=\sum_{i=1}^{n}{coef}_{i}*x_{i}$$

where $coef_{i}$ denotes the regression coefficient and $x_{i}$ denotes the expression value of each ARlncRNAs. We calculated risk scores for each STAD patient based on optimal modeling [27]. STAD patients were categorized into low-risk and high-risk groups according to the median risk score derived from the training group.

### 4.3. Analysis of Prognostic Features

The “survival” and “survminer” software packages were used to evaluate the survival rate. The survival rates of high-risk and low-risk patients were computed and shown by Kaplan–Meier survival curves. The significance level for the Kaplan–Meier analysis was determined by using a log-rank test with a threshold of *p* < 0.05. The reliability of the risk scoring model was verified by observing the risk curve, survival status distribution graph, and ROC curve. The accuracy of the prognostic model was evaluated by calculating the area under the ROC curve (AUC) using the “timeROC” software package. An AUC ≥ 0.70 under the ROC curve is usually significant, while an AUC ≥ 0.80 is excellent. An independent prognostic analysis and nomograms were used to assess the prognostic significance.

### 4.4. Methylation Analysis

STAD patients were categorized into hypermethylated and hypomethylated groups based on the median methylation values at the CpG sites of each differentially expressed ARlncRNAs. A survival difference analysis and a methylation analysis were conducted for the high- and low-methylated groups, and the correlation between the methylation level of each CpG site and the expression of LINC00511 was calculated. The “ggpubr” and “ggplot2” packages were used for drawing correlation diagrams.

The DNA methylation data (Illumina Infinium Human Methylation 450 K) and human genome annotation files of human STAD tissue samples and adjacent normal tissue samples were downloaded from the UCSC Xena (http://genome.ucsc.edu/, accessed on 5 March 2024) database. The promoter methylation sites of 18 differentially expressed ARlncRNAs were analyzed by using Perl (https://www.perl.org/, accessed on 24 October 2023) scripts, of which 2 ARlncRNAs had unnormal CpG sites in their promoter regions.

### 4.5. Chi-Squared Test

The chi-square test is employed to determine if the discrepancy between observed and expected frequencies is statistically significant [28]. The chi-square test was used to calculate the frequency of abnormal expression and methylation in different clinical characteristics groups. The basic formula for the chi-square test is as follows:

(2) $$X^{2}=\sum_{i=1}^{k}\frac{{\left(A_{i}-T_{i}\right)}^{2}}{T_{i}}$$

where $A_{i}$ is the actual frequency of aberrant methylation sites in clinical characteristics, $T_{i}$ is the theoretical frequency, and X is the chi-square value. The *p*-value of the test is obtained after the chi-square test is performed on the data in the frequency table. This *p*-value is used to judge the significance of the test results. We also used the same methodology to assess potential differences in the expression of methylation sites across various clinical characteristics.

### 4.6. Target Gene Prediction

Through the co-expression analysis between the LINC00511 and mRNA, we have identified the mRNAs that are potentially targeted by LINC00511 [29]. The mRNAs with |R| > 0.4 and *p* < 0.001 were deemed to be the mRNAs targeted by LINC00511. The starBase (https://starbase.sysu.edu.cn/, accessed on 25 March 2024) online database was utilized to predict target gene miRNAs that might bind to LINC00511.

### 4.7. Statistical Analysis

R software version 4.2.3 is a crucial tool for data processing, with the utilization of the “ClusterProfiler” package and the “org.Hs.eg.db” package for conducting a functional enrichment analysis. Without a special explanation, the difference was deemed statistically significant if the *p* < 0.05, and all *p* values were two-tailed.

## 5. Conclusions

In this study, we established a risk scoring model using lncRNAs by integrating transcriptome data from STAD cases in the TCGA database with angiogenesis-related genes, and the risk scoring model has a great prognostic ability. We identified an important gene (LINC00511) associated with STAD and validated it in an external validation set. In addition, we identified two key DNA methylation sites (cg14901671 and cg27492584) within LINC00511, both of which were negatively correlated with the expression of LINC00511. Our findings will be helpful for the pathogenesis of stomach adenocarcinoma and the study of epigenetic modification target drugs.

## Data Availability

Publicly available datasets were analyzed in this study. DNA methylation data, gene expression data, and clinical data were from the TCGA (https://cancergenome.nih.gov/, accessed on 23 October 2023), and the genomic data were from the UCSC (http://genome.ucsc.edu/, accessed on 23 October 2023). Angiogenesis-related gene data were from MSigDB (http://software.broadinstitute.org/gsea/msigdb/, accessed on 23 October 2023) and GeneCard (https://www.genecards.org/, accessed on 23 October 2023).

## Supplementary Materials

The following supporting information can be downloaded at https://www.mdpi.com/article/10.3390/ijms26052132/s1.

## Abbreviations

The following abbreviations are used in this manuscript:

SC	stomach cancer
STAD	stomach adenocarcinoma
lncRNA	long non-coding RNA
ARgene	angiogenesis-related gene
ARlncRNA	angiogenesis-related long non-coding RNA
KM	Kaplan–Meier
ROC	receiver operating characteristic curve
AUC	area under the curve
OS	overall survival
GCA	gastric cardia adenocarcinoma
miRNA	microRNA
GO	Gene Ontology
KEGG	Kyoto Encyclopedia of Genes and Genomes
