# Supplementary material for: The Methylation and Expression of LINC00511, an Important Angiogenesis-Related lncRNA in Stomach Adenocarcinoma
Source: Int J Mol Sci. 2025 Feb 27;26(5):2132. doi: 10.3390/ijms26052132 (PMC11900454; doi:10.3390/ijms26052132)
Supplement: Supplementary file 1 [file ijms-26-02132-s001.zip › ijms-3458388-supplementary.pdf]

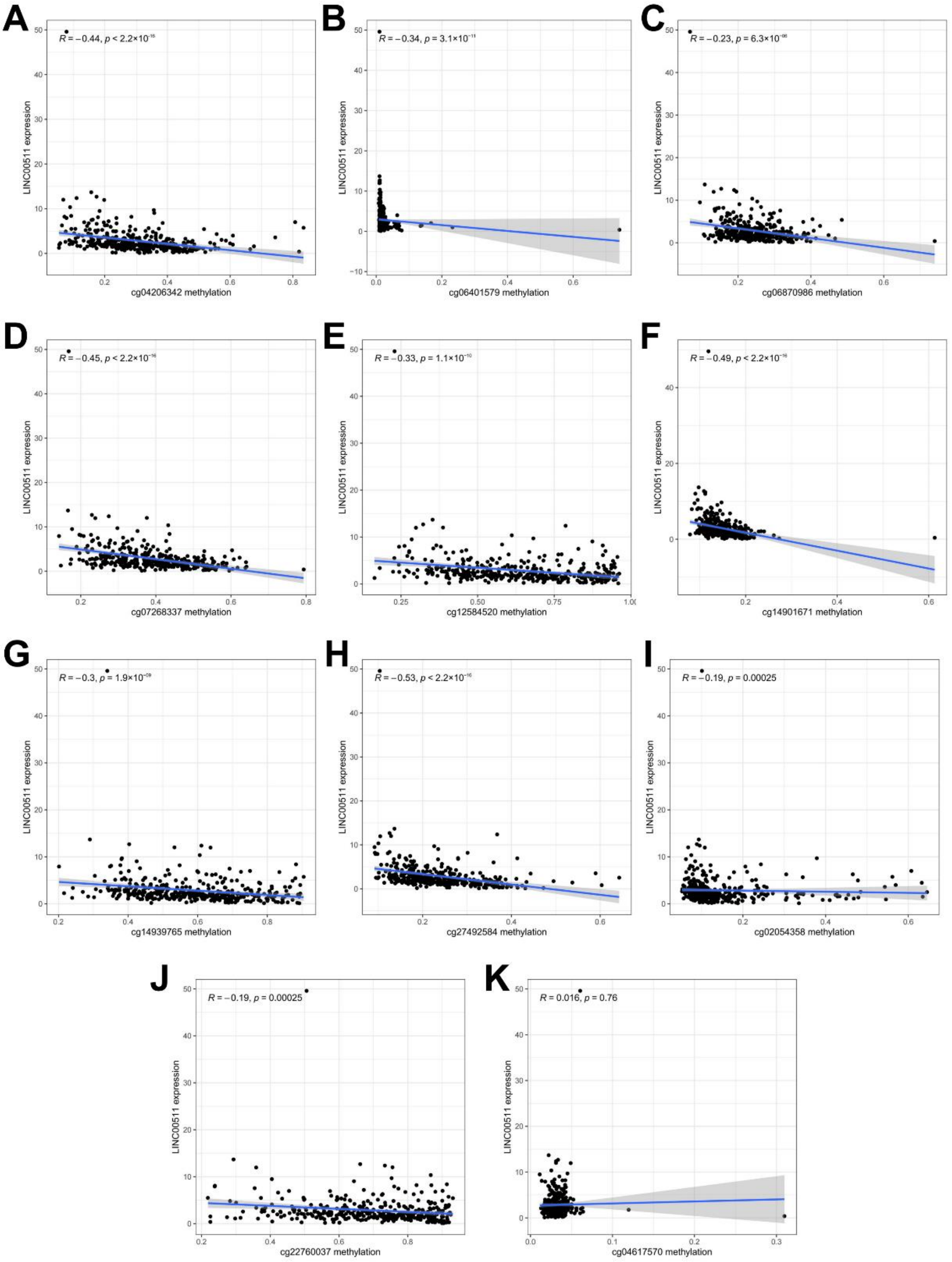

Figure S1. The relationship between the degree of methylation and expression of LINC00511. (**A–J**) The degree of site methylation is negatively correlated with the expression of LINC00511; (**K**) The degree of methylation of cg04617570 is positively correlated with the expression of LINC00511.

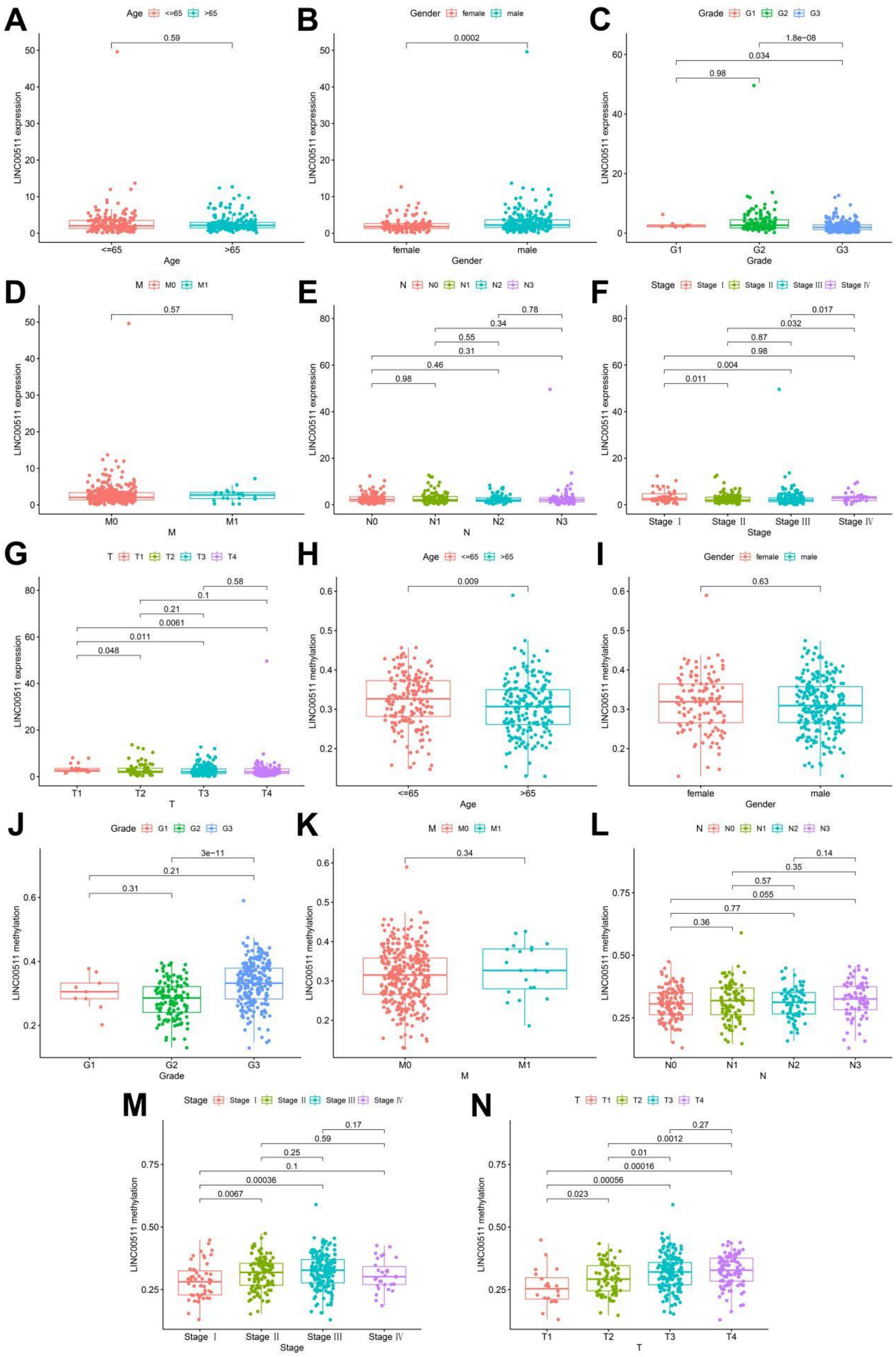

Figure S2. Clinical correlation analysis. (**A–G)** The expression of LINC00511 among different clinical groups; (H–N) Methylation of LINC00511 between different clinical groups.
